# A network meta-analysis of the efficacy and side effects of udca-based therapies for primary sclerosing cholangitis

**DOI:** 10.18632/oncotarget.5610

**Published:** 2015-09-10

**Authors:** Gui-Qi Zhu, Ke-Qing Shi, Gui-Qian Huang, Li-Ren Wang, Yi-Qian Lin, Martin Braddock, Yong-Ping Chen, Meng-Tao Zhou, Ming-Hua Zheng

**Affiliations:** ^1^ Department of Infection and Liver Diseases, Liver Research Center, The First Affiliated Hospital of Wenzhou Medical University, Wenzhou, China; ^2^ School of the First Clinical Medical Sciences, Wenzhou Medical University, Wenzhou, China; ^3^ Institute of Hepatology, Wenzhou Medical University, Wenzhou China; ^4^ Renji School of Wenzhou Medical University, Wenzhou China; ^5^ Global Medicines Development, AstraZeneca R&D, Loughborough, United Kingdom; ^6^ Department of Hepatobiliary Surgery, The First Affiliated Hospital of Wenzhou Medical University, Wenzhou, China

**Keywords:** primary sclerosing cholangitis, intervention, adverse events, clinical efficacy, network meta-analysis, Pathology Section

## Abstract

**Objectives:**

Therapies for treatment of patients with primary sclerosing cholangitis (PSC) include administration of ursodeoxycholic acid (UDCA) alone, or combination with metronidazole (MTZ) or mycophenolate mofetil (MMF), respectively. However, the optimum regimen still remains inconclusive. We aimed to compare interventions in terms of patient mortality or liver transplantation (MOLT), progression of liver histological stage (POLHS), serum bilirubin, alkaline phosphatase (ALP) levels and adverse events (AE).

**Methods:**

We searched PubMed, Embase and the Cochrane Library for randomized controlled trials until 31, Jan 2015. We estimated hazard ratios (HRs), odds ratios (ORs) and mean difference (MD) between treatments on clinical outcomes. Sensitivity analyses based on the dose of UDCA, quality of trials or treatment duration were also performed.

**Results:**

Ten RCTs were included. Compared with UDCA plus MTZ, UDCA (HR 0.28, 95%CI 0.01-3.41), UDCA plus MMF (HR 0.08, 95%CI 0.00-4.18), or OBS (HR 0.28, 95%CI 0.01-3.98) all provided an increased risk of MOLT. UDCA provided a significant reduction in bilirubin and ALP levels compared with OBS (MD −13.92, *P* < 0.001; MD −484.34, *P* < 0.001; respectively). With respect to POLHS, although differing not significantly, UDCA plus MTZ had a tendency to improve LHS more than UDCA (OR 1.33), UDCA plus MMF (OR 3.24) or OBS (OR 1.08). Additionally, UDCA plus MTZ (MD −544.66, *P* < 0.001) showed a significant reduction in ALP levels compared with OBS, but appeared to be associated with more AEs compared with UDCA (OR 5.09), UDCA plus MMF (OR 4.80) or OBS (OR 7.21).

**Conclusions:**

MTZ plus UDCA was the most effective therapy in survival rates and liver histological progression.

## INTRODUCTION

Primary sclerosing cholangitis (PSC) is a progressive cholestatic liver disease of unknown etiology characterized by inflammation and fibrosis of the intra and extrahepatic bile ducts [1]. In most cases, the disease progresses to cirrhosis, portal hypertension and liver failure within one to two decades of diagnosis [2–3]. Currently, there is no specific medical treatment for PSC that halts or reverses disease progression [2]. For patients with end-stage PSC, medical therapy offers few benefits and liver transplantation remains the ultimate treatment [4]. There is now evidence that PSC may recur in the liver allograft [4–6] [7], and liver transplantation can no longer be regarded as definitive treatment.

Ursodeoxycholic acid (UDCA), a hydrophilic bile acid, which is an effective treatment of primary biliary cirrhosis, has also been investigated as a potential candidate for the treatment of patients with PSC. However, the data regarding its clinical benefit are conflicting. Recently, four comprehensive traditional meta-analyses including eight randomized controlled studies (RCTs) concluded that although the use of UDCA has been shown to improve liver biochemistry, its effect on liver histology, prognosis and survival are inconclusive [8–11]. In addition, its effect on symptoms and quality of life are also controversial [12].

PSC patients have been offered adjuvant therapy with several immunosuppressant agents or antibiotics, various treatment strategies have been evaluated and negative studies have been published using glucocorticoids, budesonide, azathioprine, colchicine, cyclosporine, D-penicillamine, methotrexate, tacrolimus, and pentoxifyllene [13–19]. Mycophenolate mofetil (MMF) is a potent immuno-suppressant now widely used in organ transplantation [20] and the utility of MMF for the treatment of PSC remains undefined. In a pilot study of MMF monotherapy given for 1 year, Angulo et al. demonstrated a small but significant decrease in alkaline phosphatase (ALP) in patients with PSC [21]. However, another RCT by Sterling et al. [22] concluded that MMF combined with UDCA does not appear to provide additional benefit compared with standard doses of UDCA alone. While for the adjuvant therapy with antibiotics, one large, randomized controlled long-term study [23] using metronidazole (MTZ) in the treatment of PSC has been published, which indicated that combining MTZ with UDCA in PSC improved serum ALP levels, but showed no statistically significant effect on disease progression as assessed via liver histology.

Due to the limited direct comparisons obtained in clinical trials, there are some controversies in determining what the optimum UDCA-based intervention (UDCA, UDCA plus MMF, UDCA plus MTZ) is for patients with PSC. Theoretically, this may be answered by conducting a very large clinical trial with multiple comparator arms. However, it is unlikely that any single trial will compare all available treatment options. On this basis, a network meta-analysis is a potential solution, as it may permit the integration of direct and indirect comparisons, allowing us to simultaneously compare several different treatments [24–26]. In doing so, our aims were to summarize a much broader evidence base and to compare the main clinical outcomes or safety profile with four major interventions (UDCA, UDCA plus MMF, UDCA plus MTZ or observation (OBS)) for patients with PSC.

## RESULTS

### Characteristics of trials and patients

**Figure 1:**
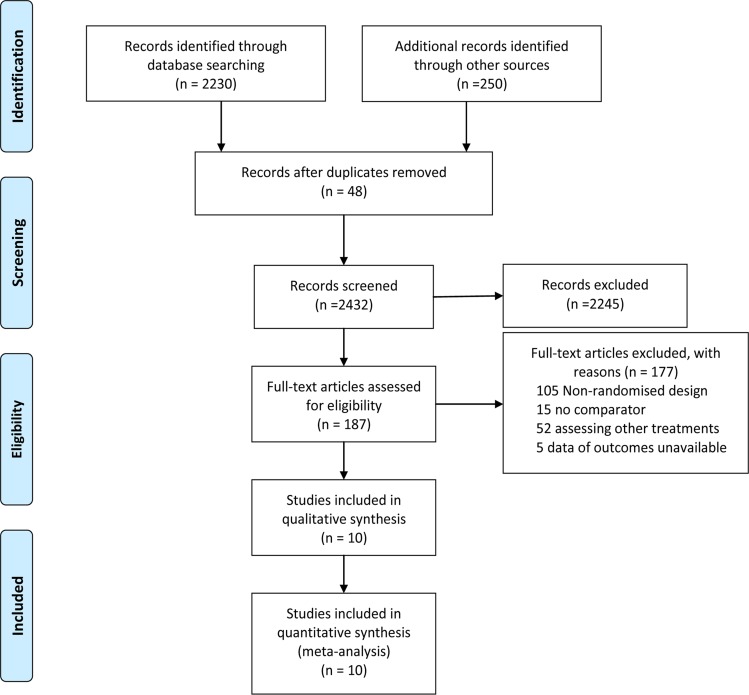
Literature search and selection

**Figure 2:**
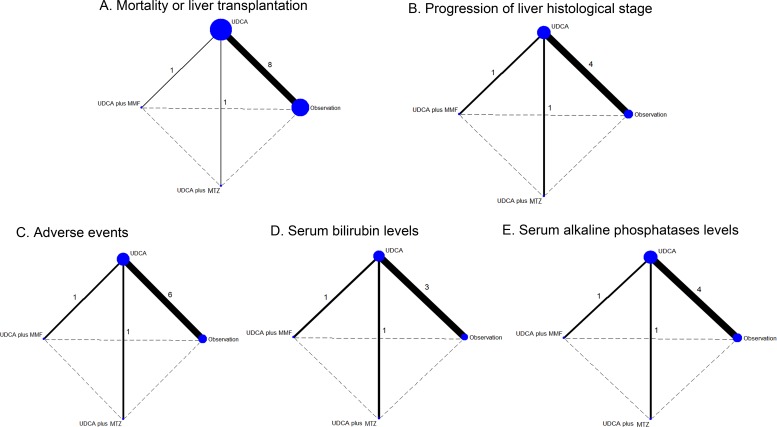
Evidence network of eligible comparisons for network meta-analysis The numbers along the link lines indicate the number of trials or pairs of trial arms. Lines connect the interventions that have been studied in head-to-head (direct) comparisons in the eligible controlled trials. The width of the lines represents the cumulative number of trials for each comparison and the size of every node is proportional to the number of enrolled participants (sample size). Different nodes referred to different interventions accordingly. UDCA: ursodeoxycholic acid; MMF: mycophenolate mofetil; MTZ: metronidazole **A.** Mortality or liver transplantation; **B.** Progression of liver histological stage; **C.** Adverse events; D. Serum bilirubin levels; **E.** Serum alkaline phosphatases levels.

Figure 1 represents the flow chart of the study and summarizes the process of identifying trials. We identified 2480 studies for review of title and abstract. After the initial screening, we retrieved the full text of potentially eligible articles for detailed assessment, 2470 articles were excluded. Ten eligible studies were included for meta-analysis, with a total of 697 patients who received one of the three treatment strategies or OBS (Figure 2). The duration of treatment ranged from three months to five years and the mean age of trial participants was 42.4 years and range from 22 to 75.6 years. Table 1 represents the characteristics of the included trials. We included 4 regimens according to eligible studies: UDCA, UDCA plus MTX, UDCA plus MMF or OBS. For the primary outcome of interest, three unique comparisons were available for ten [22–23, 27–34] different trials in mortality or liver transplantation (MOLT), six trials [22–23, 27–29, 31] in progression of liver histological stage (POLHS). In terms of adverse events (AEs), there were eight trials [22–23, 27, 29, 31–34] providing data for three unique comparisons. While for serum bilirubin and ALP, both three comparisons were analyzed from five [22–23, 29, 31, 34] and six [22–23, 27, 29, 31, 34] trials, respectively. The trials included were all two-grouped and the mean study sample was 34.9 patients per group (minimum-maximum 6-110). Quality evaluation was evaluated by the Cochrane Risk of Bias tool (Supporting information 2). Appropriate methods of random sequence generation were described for six trials (60%). Six (60%) and ten trials (100%) reported blind participants and clinical outcomes, respectively. In general, trials were considered to be of high methodological quality.

**Table 1:** Characteristics of included studies

Author (Year)	Country	Mean age (range)	Treatment (dose)	Treatment duration	Study size	Mortality or liver transplantation (%)	Adverse events (%)	Progression of liver histological stage (%)	Serum bilirubin levels (Mean/SD)		Serum alkaline phosphatases levels (Mean/SD)	
						Treatment/Control	Treatment/Control	Treatment/Control	Treatment	Control	Treatment	Control
Beuers (1992)	Germany	36 (24–44)	UDCA (13 to 15 mg/kg body weight/day in two divided doses)	1 year	14	17/25	33/13	17/38	NR/NR	NR/NR	351.2/351.76	667.14/387.33
de Maria (1996)	United States	32 (27–37)	UDCA (300 mg twice a day)	2 years	40	5/5	NR/NR	NR/NR	NR/NR	NR/NR	NR/NR	NR/NR
Lindor (1997)	United States	41.7 (39.7–43.7)	UDCA (13 to 15 mg/kg body weight/day in four divided doses)	2 years	105	7.5/5.8	2/2	13/6	25.5 /35.7	44.2 /62.9	655/481	1185/852
Lindor (2009)	United States	47.9 (20.5–75.6)	UDCA (28 to 30 mg/kg body weight/day in divided doses)	2 years	150	6.6/4.1	NR/NR	NR/NR	NR/NR	NR/NR	NR/NR	NR/NR
Lo (1992)	United Kingdom	47 (23–58)	UDCA (10 mg/kg body weight/day)	2 years	18	13/10	13/10	NR/NR	NR/NR	NR/NR	NR/NR	NR/NR
Mitchell (2001)	Germany	52 (22–79)	UDCA (20 mg/kg body weight/day in two divided doses)	2 years	26	8/15	8/8	25/25	16 /10	24/19	455/337	875/994
Olsson (2005)	Denmark	43.6 (31.6–55.6)	UDCA (17–23 mg/kg body weight/day in two divided doses)	5 years	219	2/3	34/31	NR/NR	NR/NR	NR/NR	NR/NR	NR/NR
Stiehl (1994)	Germany	36 (18–58)	UDCA (750 mg/day)	3 months	20	10/10	30/10	5/5	10.2 /1.7	25.5 /6.8	255.2/36.2	768.6/125.1
Farkkila (2004)	Finland	41 (16–65)	UDCA (15 mg/kg/day); MTZ (600 to 800 mg/day)	3 years	80	7/3	19/53	31/38	15.0 /7.3	13.5 /6.4	313/200.7	253/198.3
Sterling (2004)	United States	48 (35–61)	UDCA (13–15 mg/kg/day); MMF (13–15 mg/kg/day)	2 years	25	33/8	8/8	8/15	1.15/0.33	2.1/1.77	6 411/288	10 519/ 593

Notes: UDCA: ursodeoxycholic acid; MMF: mycophenolate mofetil; MTZ: metronidazole; NR: not reported; SD: standard deviance

### Results from pair-wise comparisons

Pairwise meta-analysis was accomplished for the three different comparisons. The weighted hazard ratios (HRs) and odd ratios (ORs) for the outcomes, MOLT, POLHS and AEs, were calculated for each comparison. While for serum bilirubin or ALP levels, each comparison was calculated as the mean difference (MD). The geometric distribution of randomized controlled trials on MOLT (Figure 2A), POLHS (Figure 2B), AEs (Figure 2C), serum bilirubin levels (Figure 2D) and ALP levels (Figure 2E) were displayed. For primary outcomes, meta-analysis of the direct comparisons did not show any significant efficacy for all interventions compared with OBS (Table 2). In the comparisons between active interventions, UDCA plus MTZ provides more benefits in reducing the risk of MOLT than UDCA (HR 3.00 95%CI 0.30 to 30.15) and UDCA plus MMF (HR 2.40 95%CI 0.19 to 30.52). Similarly, for the outcome of POLHS, UDCA (OR 0.73 95%CI 0.27 to 2.01) and UDCA plus MMF (OR 0.5 95%CI 0.04 to 6.35) may experience more in increasing LHS than UDCA plus MTZ. These results arise from 6 independent analyses. For secondary outcomes, UDCA had a significant reduction in both serum bilirubin (MD 14.64, 95%CI 10.58 to 18.70, *P* < 0.001) when compared with OBS. In addition, when compared with UDCA plus MTZ, although differing not significantly, UDCA (MD 1.5, 95%CI −1.5 to 4.5) and UDCA plus MMF (MD 0.95, 95%CI −0.18 to 2.08) did not show any reduction in serum bilirubin levels. Similarly, all the direct comparisons showed that UDCA decreased the ALP levels significantly than OBS (MD 506.24, 95%CI 429.55 to 582.93, *P* < 0.001), but with the exception of UDCA (MD −60.0, 95%CI −147.45 to 27.45) and UDCA plus MMF (MD −108.0, 95%CI −541.81 to 325.81) compared with UDCA plus MTZ. For AEs, the OR was 0.21 (95% CI 0.07-0.60, *P* < 0.001) for the comparison (UDCA plus MTZ vs. UDCA), which suggested that UDCA plus MTZ yielded a significant superior safety profile when compared with UDCA. In addition, UDCA plus MTZ, although not differing significantly, was associated with more safety profile than UDCA plus MMF (OR 0.91 95%CI 0.05 to 16.7) or OBS (OR 0.82 95%CI 0.49 to 1.36).

**Table 2:** Assessment of heterogeneity and publication bias for direct comparisons and comparison of outcomes between pair-wise meta-analysis and network meta-analysis

Treatment comparisons	Results of pair-wise meta-analysis	*I^2^* (%)	Results of network meta-analysis
Mortality or liver transplantation			
OBS vs UDCA	0.97 (0.46, 2.02)	0.0	0.98 (0.42, 2.18)
UDCA vs UDCA plus MTZ	3.00 (0.30, 30.15)	NA	3.63 (0.29, 117.67)
UDCA plus MMF vs UDCA	2.40 (0.19, 30.52)	NA	2.85 (0.16, 94.68)
Serum bilirubin			
OBS vs UDCA	14.64 (10.58, 18.70)	16.7	13.92 (0.16, 26.15)
UDCA vs UDCA plus MTZ	1.50 (−1.50, 4.50)	NA	1.53 (−18.34, 20.94)
UDCA plus MMF vs UDCA	−0.95 (−2.08, 0.18)	NA	−0.87 (−20.12, 17.89)
Serum alkaline phosphatases			
OBS vs UDCA	506.24 (429.55, 582.93)	96.3	484.34 (203.97, 709.54)
UDCA vs UDCA plus MTZ	60.0 (−27.45, 147.45)	NA	59.37 (−349.95, 469.88)
UDCA plus MMF vs UDCA	−108.0 (−541.81, 325.81)	NA	−76.87 (−673.05, 472.65)
Progression of liver histological stage			
OBS vs UDCA	1.29 (0.38, 4.35)	34.2	1.27 (0.40, 4.58)
UDCA vs UDCA plus MTZ	0.73 (0.27, 2.01)	NA	0.75 (0.12, 4.40)
UDCA plus MMF vs UDCA	0.50 (0.04, 6.35)	NA	0.41 (0.01, 8.75)
Adverse events			
OBS vs UDCA	0.82 (0.49, 1.36)	0.0	0.71 (0.23, 1.66)
UDCA vs UDCA plus MTZ	0.21 (0.07, 0.60)	NA	0.20 (0.03, 1.15)
UDCA plus MMF vs UDCA	1.09 (0.06, 19.63)	NA	1.08 (0.02, 48.52)

Notes: UDCA: ursodeoxycholic acid; MTZ: Metronidazole; MMF: mycophenolate mofetil; OBS: observation; NA: not available;

Due to the limited availability of RCTs in some comparisons (UDCA versus UDCA plus MTZ, UDCA versus UDCA plus MMF), we are unable to assess statistical heterogeneity for those pair-wise comparisons. Overall, for the direct comparison of UDCA versus OBS, statistical heterogeneity was moderate (Table 2). In the meta-analyses of direct comparison (UDCA versus OBS) for primary outcomes, I² values lower than 50% were recorded both in MOLT (0.0%) and POLHS (34.2%). While for secondary outcomes, I² values lower than 50% were recorded in serum bilirubin concentration (16.7%), with the exception of comparisons UDCA versus OBS (96.3%) in serum ALP levels. Similarly, I² values lower than 50% was also recorded in AEs (0.0%).

### Results from the network meta-analysis of primary and secondary outcomes

**Figure 3:**
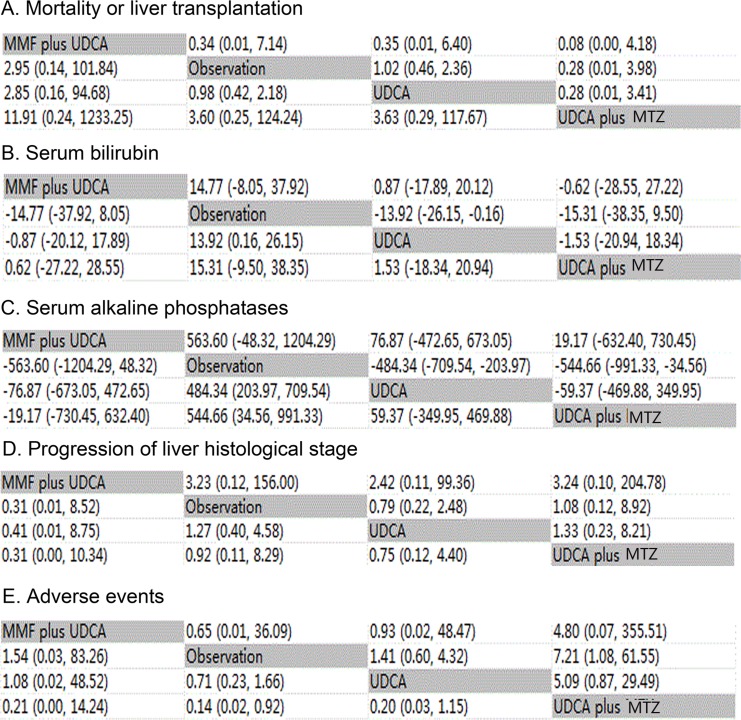
Major clinical efficacy and safety of all treatments according to network meta-analysis Treatments are reported in alphabetical order. The ORs were estimated in upper and lower triangle comparing column-defining with row-defining treatment. For clinical improvement, ORs higher than 1 favor the column-defining treatment, while for adverse effects, ORs lower than 1 favor the row-defining treatment. Similarly, for blood ammonia concentration and mental status, MDs lower than 0 favor the column-defining treatment. UDCA: ursodeoxycholic acid; MMF: mycophenolate mofetil; MTZ: metronidazole **A.** Mortality or liver transplantation; **B.** Serum bilirubin levels; **C.** Serum alkaline phosphatases levels; **D.** Progression of liver histological stage; **E.** Adverse events.

The HRs and ORs for MOLT, POLHS and AEs respectively, and MD for serum bilirubin and ALP levels with 95% confidence intervals obtained from the indirect comparisons of the included regimens are showed in Figure 3. As the network framework displayed, compared with OBS in terms of primary outcomes, although not differing significantly, UDCA was associated with higher risk in causing MOLT (HR 0.98, 95%CI 0.42 to 2.18). While for the outcome of POLHS, UDCA confer more benefits in decreasing LHS (OR 1.27, 95%CI 0.40 to 4.58). In the comparisons between active interventions, UDCA plus MTZ provided more benefits in the reduction of MOLT or POLHS than UDCA (HR 0.28, 95%CI 0.01 to 3.41); (OR 0.75, 95%CI 0.12 to 4.40), UDCA plus MMF (HR 0.08, 95%CI 0.00 to 4.18); (OR 0.31, 95%CI 0.00 to 10.34) or OBS (HR 0.28, 95%CI 0.01 to 3.98); (OR 0.92, 95%CI 0.11 to 8.29).

For our assessment of serum bilirubin as discontinuous outcomes (Figure 3B), UDCA (MD −13.92, 95%CI −26.15 to −0.16) appears to show a statistically significant effect in reducing serum bilirubin levels when compared with OBS. However, statistical significance was not reached for other comparisons, UDCA plus MTZ appeared to demonstrate a greater reduction in serum bilirubin levels than UDCA (MD −1.53, 95%CI −20.94 to 18.34), UDCA plus MMF (MD −0.62, 95%CI −28.55 to 27.22) or OBS (MD −15.31, 95%CI −38.35 to 9.5). With respect to serum ALP levels, compared with OBS, UDCA (MD −484.34, 95%CI −709.54 to −203.97) and UDCA plus MTZ (MD −544.66, 95%CI −991.33 to −34.56) showed a statistically significant reduction in ALP concentration. However, no statistical significance was demonstrated for other comparisons. UDCA plus MMF tended to demonstrate a greater reduction in blood ALP levels than UDCA (MD −76.87, 95%CI −673.05 to 472.65), UDCA plus MTZ (MD −19.17, 95%CI −730.45 to 632.40) or OBS (MD −563.60, 95%CI −1204.29 to 48.32).

In the assessment of adverse event outcomes (Figure 3E), a total of 253 (49.7%) patients were specifically assigned to UDCA treatment, 39 (7.7%) to UDCA plus MTZ treatment, 12 (2.4%) to UDCA plus MMF treatment. In addition, 204 (40.1%) patients were randomized to OBS. When compared with OBS, therapy with UDCA plus MTZ (OR 7.21, 95%CI 1.08 to 61.55, *P* < 0.001) was associated with significant adverse effects. However, other comparisons among treatments showed no statistical significance in AEs. UDCA plus MTZ appeared to show more AEs in comparison with UDCA (OR 5.09, 95%CI 0.87 to 29.49) and UDCA plus MMF (OR 4.80, 95%CI 0.07 to 355.51).

**Figure 4:**
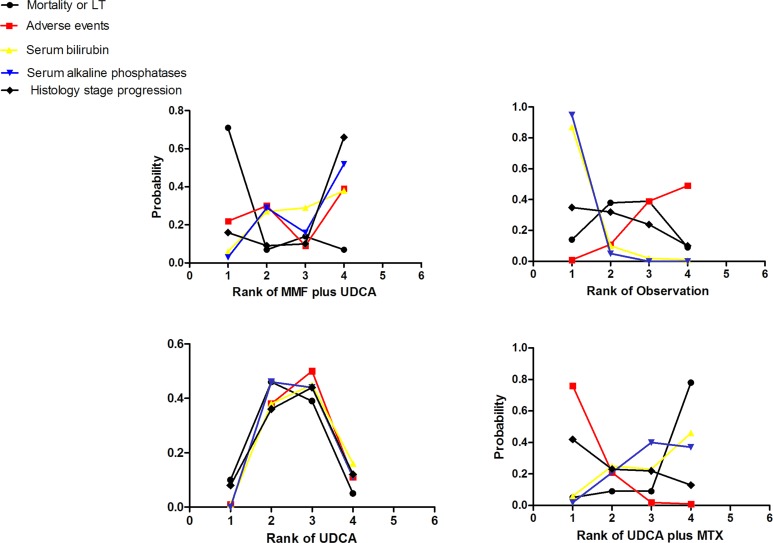
Rankograms showing probability of each strategy having each specific rank (1-4) for mortality or liver transplantation, adverse events, serum bilirubin and alkaline phosphatases levels Ranking indicates the probability to be the best treatment, the second best, the third best and so on. Rank 1 is best and rank N is worst. UDCA: ursodeoxycholic acid; MMF: mycophenolate mofetil; MTZ: metronidazole.

Finally, we ranked the likelihood of best treatment for each intervention at each of the 4 possible parameters (Figure 4). UDCA plus MTZ (78%) showed the highest likelihood of reduction in MOLT (Figure 4), suggesting UDCA plus MTZ was more efficacious than the other remaining interventions. Consistently, UDCA plus MTZ (42%) and UDCA (44%) showed a greater probability of being the two most effective interventions with respect to a decreasing in patient LHS, suggesting that UDCA plus MTZ and UDCA were more efficacious than the other interventions. In terms of serum bilirubin and ALP levels, UDCA plus MTZ (46%; 37%) and UDCA plus MMF (38%; 52%) reduced bilirubin or ALP levels in blood better than the other remaining interventions. However, UDCA plus MTZ (76%) ranked the highest intervention with respect to AEs and our ranking suggests that interventions with the safest effects were UDCA (50%) and OBS (49%). Supporting Information 3 presents a comparison-adjusted funnel plot for the interventions network (limited trials for serum bilirubin, POLHS and ALP levels), without evidence of asymmetry, which suggests the absence of small-study effects.

### Sensitivity analysis

We conducted several of the planned sensitivity analyses which are regarding duration of treatment (Supporting Information 4a), quality of trials (Supporting Information 4b) and dose of UDCA administered (Supporting Information 4c). The summary of included or excluded studies in all three sensitivity analyses is showed in Supporting Information 5. We defined short treatment duration as being less than 24 months and long treatment duration as being 24 months or longer. We also defined a low dose of UDCA (less than 13 mg/kg body weight/day) and a high dose (13 mg/kg body weight/day or more) by the median dose of UDCA used in the trials included in this analysis. There were eight trials [22–23, 28–33] included which reported long treatment duration, five trials [22–23, 27, 29,31] included which patients administrated by high dose of UDCA and eight trials [22–23, 27, 29–33] included which reported low or moderate risk of study quality in our sensitivity analyses. Overall, results closely resembled our primary network meta-analysis with similar effect estimates and rankings for all three sensitivity analyses. Supporting Information 4a, 4b, 4c indicates that combination of MTZ and UDCA was the top-ranked treatments for both the outcomes of MOLT or POLHS in all three sensitivity analyses, although most indirect comparisons do not differ significantly. Consistently, for secondary outcomes in all three sensitivity analyses, UDCA plus MTZ and UDCA plus MMF was the top-ranked treatments in reducing serum bilirubin or ALP levels, respectively. Similar findings were also observed for the outcome of AEs. UDCA plus MTZ was associated with more AEs than other UDCA-based treatments, similarly, UDCA and OBS was ranked the least possible regime to cause AEs.

### Model fit and consistency of the network

The model fit can be evaluated using the posterior mean of the residual deviance (D res), we calculated the values of the D res for all outcomes, which were close to corresponding number of data points for the outcomes, meaning that model’s overall fit is relatively satisfactory (Supporting Information 6). The results of traditional pairwise and network meta-analyses were also showed in Table 2. The confidence intervals from both the traditional pairwise meta-analyses and the Bayesian network meta-analyses, although the pooled estimates showed small differences, are in general consistently compatible.

## DISCUSSION

In the absence of head-to-head comparisons, we performed a network meta-analysis that evaluates the efficacy and safety of current UDCA-based treatments available in RCTs for PSC, including two combination regimens and one monotherapy with UDCA. Our study found that MTZ plus UDCA was the most effective in reducing the risk of MOLT and POLHS, but was associated with an increased adverse effect profile. Although UDCA provided little further survival benefit, it showed a safer AE effect profile than other UDCA combination interventions. Additionally, our synthesis of the available studies demonstrated that all regimes had greater efficacy than OBS for biomedical variables outcomes (serum bilirubin and ALP levels). Specifically, a combination of MTZ and MMF with UDCA, respectively, were the most beneficial for the reduction of blood bilirubin and ALP levels for patients with PSC.

Our study has several strengths. The internal validity of our analysis is supported by three factors. First, having conducted a rigorous and extensive literature search, we are confident that all relevant RCTs have been properly identified. Secondly, the most trials included in the network are characterized by low risk of bias, assessed by Cochrane Collaboration approach and allowed a reliable synthesis of Bayesian indirect treatment effect estimates. Thirdly, most RCTs are conceptually homogeneous in terms of study design and patient characteristics. Even though differences in key characteristics (dose of UDCA administered, treatment duration) across trials that may affects the synthesis of results, we therefore performed several sensitivity analyses and finally find similar results closely resembled the results presented in the primary network meta-analysis with similar effect estimates and rankings. Besides, the overall model fit for five outcomes is relatively satisfactory and inconsistency of network seems small.

However, the strengths of this network meta-analysis should be weighed against some limitations. First, the limited number of trials and the absence of head-to-head comparisons may increase the uncertainty of the findings and conclusions.

Secondly, the indirect estimates were often very similar to those obtained in the direct comparisons because only single comparisons were available for the majority of the cases. This resulted in a less conventional geometry where our network of trials did not have any closed loops. Thirdly, we could not assess publication bias for most comparisons. Finally, our analysis was that not all outcomes of interest were reported consistently across trials. In general, missing data resulted in wider confidence intervals due to greater uncertainty around the estimates. However, despite of these limitations, this network meta-analysis provides the largest scale comparative information on the major clinical outcome profiles of different interventions in current use.

UDCA is an effective treatment of primary biliary cirrhosis and has been investigated as a potential candidate for the treatment of PSC. There were different doses of UDCA used in the included trials in this meta-analysis. Three RCTs used a low dose of UDCA (8.5-15 mg/kg daily), while four RCTs used a higher dose of UDCA (17-23 mg/kg and 28-30 mg/kg). The effects of UDCA has been demonstrated by several studies [27–33], which consistently concluded that it provided no clinical benefits for patients with PSC, except that one RCT published in 2001 [31] showed that high dose UDCA may be of clinical benefit. However, it was difficult to detect any survival benefit for high dose UDCA because of the small number of patients and the relatively short follow-up period of 2 years. Hence, one possible explanation for inconsistent results was that the small patient population and a larger number of participants and of longer duration is required. In addition, several pair-wise meta-analyses reached the same conclusions as we had showed in our analysis. Five published traditional meta-analysis [8–11, 35] all reported that neither high nor low dose UDCA had beneficial effect on patient survival, which are consistent with our results. Overall, a higher risk of MOLT with UDCA compared to the OBS group was achieved in our meta-analysis. One possibility is that UDCA is simply not effective in PSC. The improvement in biochemical values may argue against this, but caution should be exercised to avoid over-interpreting biochemical improvement without detectable clinical improvement. Another possibility is that UDCA is not sufficiently well absorbed to be able to exert a beneficial effect. However, in terms of liver biomedical variables, consistently, three traditional meta-analyses [9–10, 35] showed that UDCA may lead to a significant improvement in serum bilirubin or ALP levels. While in terms of adverse events, long-term UDCA administration in patients with PSC was associated with a very low incidence of adverse events in the six included trials; these findings confirm previous observations in patients with primary biliary cirrhosis and viral hepatitis [36–37]. The major AE reported in these trials was diarrhea. Overall, as presented in network meta-analysis, we utilized the largest data on patients administrated by UDCA, and furthermore, our results are robust as sensitivity analyses showed resemble results with our major outcomes.

Our study also evaluated the effects of combined MTZ and MMF with UDCA. One RCT published by Sterling et al. [22] showed that MMF plus UDCA does not appear to provide additional survival benefit compared with standard doses of UDCA alone. However, compared with UDCA, MMF plus UDCA was well-tolerated and no patient developed worsening diarrhea, abdominal pain or episode of cholangitis, which we also found in both our direct and indirect comparisons. Similarly, another RCT [23], included in our meta-analysis concluded that MTZ plus UDCA in PSC improved serum ALP levels significantly, whereas no statistical significance in POLHS can be seen. While for AEs, patients on UDCA/MTZ had significantly more adverse effects (53%) than patients on UDCA monotherapy. However, they were mostly mild, and none of them was severe enough to require cessation of therapy.

In summary, our analysis shows the superiority of using UDCA plus MTZ treatment in clinical efficacy for patients with PSC, but should weigh its increased AEs. The analysis also provides indirect evidence that all interventions are better than OBS in decreasing blood bilirubin or ALP levels. Combing MTZ or MMF respectively with UDCA were the most effective treatments in terms of serum bilirubin and ALP levels. Direct head-to-head comparisons between UDCA-based interventions should be a priority on the research agenda, as well as large number participants and evaluation in long term follow up, which is a key consideration in determining the comparative effectiveness of treatments for PSC.

## MATERIALS AND METHODS

### Search strategy

This systematic review is reported according to the PRISMA (Preferred Reporting Items for Systematic Reviews and Meta-Analyses) guideline (Supporting information 1) [38]. We searched four electronic databases (PubMed, Embase, and the Cochrane Library) up to 31 Jan 2015 for randomized controlled trials investigating UDCA-based interventions for patients with PSC using the key terms “primary sclerosing cholangitis, ‘ursodeoxycholic acid and treatment” without any language or date restrictions. The bibliographies of selected articles were searched in an effort to identify any other relevant articles. Two reviewers (Gui-Qi Zhu, Ke-Qing Shi) independently assessed the eligibility of all potential abstracts and titles. The study was approved by the Ethics Committee of the First Affiliated Hospital of Wenzhou Medical University.

### Selection criteria

Studies included fulfilled the following criteria: (i) there was a randomized design irrespective of blinding; (ii) patients with a diagnosis of PSC documented by cholangiography; (iii) interventions: UDCA-based or was compared with placebo or no intervention. (iv) one or more of the following outcomes were assessed: MOLT, AEs, serum bilirubin, ALP and POLHS. The flow diagram of the studies excluded from this analysis is shown in Figure 1. Eligible studies had to be published as full length articles in peer reviewed journals. Other exclusions were trials that comprised a non-randomized design, no comparator, studies comparing other therapies or data of outcomes unavailable. In addition, trials with patients with co-existing ulcerative colitis and on treatment for that were also excluded.

### Data extraction and quality assessment

Two reviewers (Gui-Qi Zhu, Ke-Qing Shi) abstracted the data independently. The following information was collected from each study: publication data; first author’s last name; geographic location of study; year of publication; study design; number of participants and population characteristics; and interventions’ variables, including duration, drug, dose, and administration, serum bilirubin and serum ALP and the number of events of interest in each group and outcomes (MOLT, POLHS and AEs). The OBS arm specifically referred to placebo or no interventions. Any discrepancies regarding the extraction of data were resolved by an additional investigator (Ming-Hua Zheng). In addition, we defined the two trials which have a common title, author and published journal as duplication, and only used the available data from one trial. When relevant information on design or outcomes was unclear, or when some needed data was unavailable directly from the study, the original authors were contacted for clarifications and assistance by email.

The quality of the methodology was independently assessed by two reviewers using the Cochrane Risk of Bias Tool. This tool includes the following items: sequence generation for the randomization of subjects, allocation of concealment of treatment, blinding, incomplete outcome data, selective outcome reporting and other sources of bias [39]. The answers for this items include (Yes, No, Unclear). Trials with high or unclear risk for bias for any one of the first three components were regarded as trials with high risk of bias. Otherwise, they were considered as trials with low risk of bias.

### Assessed outcomes

The outcome measures reported by most trials were mortality, histological changes, biochemical variables, and AEs. The primary outcome of interest was the relative efficacy of different UDCA-based interventions for PSC in reducing MOLT, decreasing histological progression. Besides, the secondary outcome of interest was the changes in biomedical variables (serum bilirubin and ALP levels) measured at the end of the intervention. In addition, to assess the safety of therapy, we also measured the relative rates of medication discontinuation as a result of AEs.

### Data analysis

We performed traditional pairwise meta-analysis using the method of DerSimonian and Laird random effects model we calculated the pooled estimates of odds ratios and 95% confidence intervals of direct comparisons between two strategies according to Cochrane Handbook for Systematic Reviews of Interventions Version 5.1.0. Publication bias was examined with the funnel plot method from pair-wise meta-analysis. I^2^ (presented as Q) were represented as markers of heterogeneity. Given that some pairwise comparison (UDCA vs UDCA plus MTZ, UDCA vs UDCA plus MMF) included a limited number of RCTs, we could not formally assess statistical heterogeneity and publication bias. We defined I^2^ values between 30% and 60% as moderate heterogeneity, 60-75% as considerable heterogeneity and values >75% as substantial heterogeneity. Values below 30% were considered unimportant [40].

Additionally, we conducted the network meta-analysis within a Bayesian framework using Markov chain Monte Carlo methods in WinBUGS (Medical Research Council Biostatistics Unit, Cambridge, United Kingdom). A network meta-analysis synthesizes all available evidence within a consistent framework, thereby fully preserving the randomization within each trial [41]. It accounts for multiple comparisons within a trial when there are more than 2 treatment groups [42–43]. As we had described in our previous published network meta-analysis [44–47], analysis was based on non-informative priors for relative-effect parameters (flat normal with mean of 0 and precision of 0.001) and between-study SD (a flat uniform distribution between 0 and 2). Convergence and lack of autocorrelation were checked and confirmed after a 5000-simulation burn-in phase without any thinning and using 4 chains with different initial values. Then, a burn-in phase of 20 000 iterations was used, followed by 50 000 iterations to estimate parameters. In order to examine the robustness of our results, we performed sensitivity analyses regarding duration of treatment, quality of trials and dose of UDCA administered by using Bayesian analytical approach.

The pooled HRs, ORs and MD from the network meta-analysis were compared with corresponding ORs or MD from pair-wise random-effects meta-analysis of direct comparisons to assess whether there was inconsistency between direct and indirect comparisons. Besides, to formally check whether a model’s overall fit was satisfactory, we considered an absolute measure of fit: D res, the posterior mean of the residual deviance (the deviance for the fitted model minus the deviance for the saturated model). We would expect that each data point should contribute about 1 to the posterior mean deviance so that can be compared to the number of data points for the purpose of checking model fit [48].

Finally, we ranked the treatments for each outcome in each simulation on the basis of their posterior probabilities. We assessed the probability that each treatment was the most effective therapy, the second best, and so on, by counting the proportion of simulations in which each treatment had the smallest ORs, the second smallest, and so on. Even though the differences in effect size among treatments obtained were small, clinical decisions about the choice of treatments can still be suggested based on the probabilities of treatment ranking. The pooled HRs for dichotomous data were reported in terms of MOLT, and ORs for POLHS and AEs, whereas serum bilirubin and ALP levels were calculated as MD with corresponding 95% confidence intervals, and as well as the probabilities of ranking by treatment. Therefore, the bayesian network meta-analysis increased statistical power by incorporating both direct and indirect evidence across all interventions.

## SUPPLEMENTARY MATERIAL



## Abbreviations

PSC: primary sclerosing cholangitis
UDCA: ursodeoxycholic acid
MTZ: metronidazole
MMF: mycophenolate mofetil
MOLT: mortality or liver transplantation
POLHS: progression of liver histological stage
ALP: alkaline phosphatase
AE: adverse events
HR: hazard ratio
OR: odds ratio
MD: mean difference
RCT: randomized controlled study
OBS: observation
CI: confidence interval
